# Visual Presentation and Factors Affecting Visual Outcome in Children with Craniopharyngioma in East Coast States of Peninsular Malaysia: A Five-year Review

**DOI:** 10.7759/cureus.4407

**Published:** 2019-04-08

**Authors:** Ismail Mohd-Ilham, Ghanimi Ramli Ahmad-Kamal, Wan-Hazabbah Wan Hitam, Ismail Shatriah

**Affiliations:** 1 Ophthalmology, School of Medical Sciences, Universiti Sains Malaysia, Kubang Kerian, MYS

**Keywords:** pediatric craniopharyngioma . visual presentation . final visual outcome

## Abstract

Purpose

To describe the visual presentation and factors affecting visual outcome in pediatric patients treated for craniopharyngioma at a referral center in the East Coast states of Peninsular Malaysia.

Methodology

A retrospective review of medical records of children aged 17 years and below who had been treated for craniopharyngioma in Hospital Universiti Sains Malaysia from January 2014 to December 2018. The data collected included age, gender, presenting symptoms and duration, visual acuity, visual fields, color vision, light brightness, relative afferent pupillary defects, fundus examination and cranial nerves examination. The best corrected visual acuity during presentation, and after a one-year post-operative period, was documented. Records on investigations, surgical procedures, therapeutic modalities and recurrences were also reviewed.

Results

A total of 11 pediatric patients (22 eyes) were recruited. Fifty percent presented with optic atrophy. The mean duration of the onset of symptoms before consultation was 22.3 (24.5) months. A final best corrected visual acuity of 6/12 (20/40) or better was observed in 50% of the patients. There was a statistically significant association between presenting visual acuity, optic nerve function and visual field defects, and the final visual outcome.

Conclusions

Visual presentations in our study were fairly similar to previous reported studies. One-third presented late with permanent visual loss. Almost half had significant visual impairment after one-year post-operative period. Significant associations were observed between presenting visual acuity, duration of symptoms, impairment of optic nerve function tests, and visual field defects during presentation, and final visual acuity at one year after treatment.

## Introduction

Craniopharyngioma is a rare intracranial tumor. It is a benign epithelial tumor originating from the remnants of Rathke’s pouch, and it is localized in either the sellar or parasellar regions [1]. The systemic sequelae and visual prognosis are strongly related to the tumor’s close proximity to surrounding vital structures. The anterior visual pathway is among the most common anatomical structure being compressed by the tumor, leading to a classical visual presentation of bitemporal hemianopia, inferior quadrantanopia and optic nerve atrophy.

The incidence rate for Western countries is estimated at 0.13 per 100,000 people annually [2]. In contrast, the annual rate for Asian countries is even higher with an estimated 0.24 to 0.53 incidents per 100,000 [3-5]. Reports on final visual outcome for patients with pediatric craniopharyngioma have been published in Europe, the Middle East and the United States [6-10].

Based on a PubMed search, the only data available on visual outcomes for Asian children with craniopharyngioma was from Korea [11-12]. This study aims to describe the visual presentation and factors affecting visual outcome for pediatric patients with craniopharyngioma in the East Coast states of Peninsular Malaysia, and to discuss the existing literature.

## Materials and methods

A retrospective survey was performed on pediatric patients (age 17 years old or younger) with craniopharyngioma who were treated at Hospital Universiti Sains Malaysia in Kelantan, Malaysia, from January 2014 to December 2018, and who received a follow-up for at least one year after treatment. This study was conducted in accordance with the Declaration of Helsinki, and the study protocol was approved by the Research and Ethical Committee, School of Medical Sciences, Universiti Sains Malaysia. Hospital Universiti Sains Malaysia is the leading referral center for the East Coast states of Malaysia for pediatric neurology and ophthalmology services.

All children diagnosed with craniopharyngioma, based on clinical, radiological and histopathological examination, were included in this study. Those with pre-existing ocular problems such as amblyopia, other causes of optic neuropathy and infiltrative diseases, were excluded.

The data collected included age, gender, presenting symptoms and duration, visual acuity, visual fields, color vision, light brightness, relative afferent pupillary defect, fundus examination and cranial nerves examination. The best corrected visual acuity during presentation, and after a one-year post-operative period, was documented.

Visual fields were tested using confrontational test or by using the Humphrey visual field test. Visual fields were described as bitemporal hemianopia, homonymous hemianopia, quadrantanopia, unilateral temporal field defect, scotoma or normal fields. Records on investigations, surgical procedures, therapeutic modalities and recurrences were also reviewed.

Visual acuity was classified as ‘good’ if the best corrected visual acuity was better than or equal to 6/12 (20/40) during presentation or at the one-year post-operative review. Data was analyzed using the Statistical Package for the Social Sciences for Windows version 24 (SPSS Inc., Chicago, IL, USA). Factors affecting the final visual outcome were further analyzed based on the Fisher exact test.

## Results

Table 1 represents clinical profile of the study population. A total of 11 pediatric patients (22 eyes) with craniopharyngioma were recruited according to the study protocol. A majority of the patients were male (63.6%), ranging in age from 3 to 16 years, with the mean age of 9.5 (4.2) years. The mean duration for the onset of symptoms before consultation was 22.3 (24.5) months, ranging from one month to five years.

**Table 1:** Clinical profile. M: Male; F: Female; BE: Both eyes; RE: Right eye; LE: Left eye; BOV: Blurring of vision; HM: Hand movement; PL: Perception of light; CF: Counting fingers; NPL: No perception of light; NA: Not available. Color vision based on Ishihara Color chart.

No	Age	Gender	Ocular symptoms	Systemic symptoms	Duration (Months)	Presenting visual acuity		Final visual acuity		Relative afferent pupillary defect	Ocular motility	Funduscopy	Optic nerve function test							
													Red saturation			Light brightness			Color vision	
						RE	LE	RE	LE				RE		LE	RE	LE		RE	LE
1	11	M	BE BOV Color defect	Clumsiness	7	6/6	HM	PL	HM	Positive	Normal	LE disc pale	100		10	100	20		15/15	0/15
2	8	F	Nil	Headache, recurrent vomiting, nocturnal enuresis	36	PL	6/12	PL	6/6	Positive	Normal	BE discs pale	0		100	50	100		0/15	15/15
3	10	M	Nil	Seizure fever	1	6/7.5	6/9	HM	NPL	Positive	Normal	BE discs temporal pallor	NA			NA			NA	
4	6	F	Nil	Frontal headache, recurrent vomiting, loss of weight and loss of appetite	24	6/7.5	6/9	6/7.5	6/9	Negative	Normal	Normal	NA			NA			NA	
5	16	F	RE BOV LE squint diplopia	Headache giddiness	72	6/6	6/6	6/6	6/6	Negative	Limited LE abduction	Normal	100	100		100	100		15/15	15/15
6	9	F	BE BOV	Headache, recurrent vomiting	5	6/120	CF	6/120	HM	Equivocal	Limited BE abduction	BE discs pale	100	50		100	50		NA	
7	3	M	Nil	Recurrent vomiting, left-sided body weakness, limping gait	4	6/24	6/15	6/7.5	PL	Positive	Normal	Normal	NA			NA			NA	
8	7	M	RE BOV BE squint	Headache, recurrent vomiting	1	HM	6/9	NPL	6/7.5	Positive	Normal	BE discs pale	NA			NA			NA	
9	5	M	Nil	Frontal headache	24	6/6	6/6	6/7.5	6/12	Negative	Normal	BE papilledema	100		100	100		70	12/15	14/15
10	14	M	Nil	Headache, recurrent vomiting	12	NPL	6/9	NPL	6/9	Positive	Normal	BE discs pale	NA			NA			NA	
11	15	M	Nil	Headache, recurrent vomiting	60	6/36	6/9	6/24	6/12	Negative	Normal	Normal	NA			NA			NA	

A majority of the patients (63.6%, n = 7) had no visual symptoms during presentation. Four patients (36.4%) presented with visual loss and two patients (18.2%) showed signs of strabismus. Nine eyes (40.9%) presented a best corrected visual acuity worse than 6/15 (20/50), while a final best corrected visual acuity of 6/12 (20/40) or better was observed in 11 patients (50.0%). Optic atrophy was documented in 11 patients (50.0%). Four patients (18.1%) manifested defects in color vision, while seven patients (31.8%) had relative afferent pupillary defects, and two patients (9.1%) exhibited evidence of papilledema on presentation. Eleven eyes (50.0%) had visual field defects. Out of these 11 eyes, the most common visual field defect was temporal hemianopia (22.7%, five eyes). Other visual field defects included central scotoma (9.1%, two eyes), inferior scotoma (4.6%, one eye), and quadrantanopia (9.1%, two eyes). We were unable to perform visual field testing on the remaining five eyes (22.7%) due to lack of cooperation. These are summarized in Table 2.

**Table 2:** Demographic and clinical data. *Calculated based on 22 eyes. SD: Standard deviation

Variables	No (%)
Total patient (n)	11
Age	
Mean (SD), years	9.5 (4.2)
Range, years	3–16
Gender	
Male	7 (63.6)
Female	4 (36.4)
Presenting visual acuity*	
6/6 - 6/12	13 (59.0)
6/15 - 6/60	3 (13.6)
Worse than 6/60	6 (27.3)
Final visual acuity*	
6/6 - 6/12	11 (50.0)
6/15 - 6/60	1 (4.5)
Worse than 6/60	10 (45.5)
Visual field defects*	
Hemianopia	
Bilateral	2 (9.1)
Unilateral	3 (13.6)
Scotoma	3 (13.6)
Quadrantanopia	2 (9.1)
Constricted visual field	1 (4.6)
Normal	6 (27.3)
Not available	5 (22.7)

Table 3 shows the investigation and treatment profile of pediatric patients with craniopharyngioma. Computed tomography (CT) scans and magnetic resonance imaging (MRI) were performed on all patients. Evidence of suprasellar masses were documented for all patients. A majority of the lesions were cystic (63.6%) and, four patients (36.4%) displayed evidence of mixed cystic and solid lesions. Most of our patients (75%) underwent a total resection of the mass, while one patient (12.5%) had the tumor biopsied.

**Table 3:** Investigations and treatment profile. NA: Not available; CT: Computed tomography; MRI: Magnetic resonance imaging; VF: Visual field; LE: Left eye; RE: Right eye.

No	Visual field on presentation					Imaging (CT scan/MRI)			Histopathology	Surgical intervention	Follow-up (months)	Recurrence
	Humphrey VF			Confrontation test								
	RE	LE		RE	LE	Location	Extension	Characteristics				
1	NA	Inferior scotoma		NA		Suprasellar	Prepontine, frontal inter-hemispheric region	Cystic calcified lobulated	NA	Refuse intervention	36	NA
2	NA			Temporal hemianopia	Temporal hemianopia	Suprasellar	Third ventricle, floor of sellar turcica	Cystic lobulated	Adamantinomatous	Bifrontal craniotomy and tumor excision	40	Yes
3	Supero-temporal quadrant-anopia		Temporal hemianopia	NA		Suprasellar	Cavernous sinus	Mixed solid and cystic	Adamantinomatous	Left pterional craniotomy with tumor debulking	16	Yes
4	Normal		Normal	Normal	Normal	Suprasellar	Nil	Mixed solid and cystic	Adamantinomatous	Vertex craniotomy with tumor debulking	8	No
5	Normal		Normal	Normal	Normal	Suprasellar	Anterior pons	Cystic calcified multi-lobulated	NA	Refuse intervention	17	NA
6	NA			Central scotoma	Central scotoma	Suprasellar	Third ventricle	Cystic	Adamantinomatous	Ventriculoperitoneal shunt with tumor debulking	20	Yes
7	NA			NA		Suprasellar	Anterior commissural, Midbrain, Pons	Cystic calcified lobulated	Adamantinomatous	Left pterional craniotomy with tumor debulking	24	Yes
8	NA			NA	Temporal hemianopia	Suprasellar	Nil	Cystic calcified	Adamantinomatous	Right pterional craniotomy with tumor debulking	24	Yes
9	NA			Normal	Normal	Suprasellar	Hypothalamic region	Mixed solid and cystic lobulated	Adamantinomatous	Ventriculoperitoneal shunt with endoscopic tumor biopsy	36	No
10	NA			NA	Temporal hemianopia	Suprasellar	Optic chiasm, pituitary fossa	Mixed solid and cystic	NA	Right supraorbital keyhole craniotomy with tumor debulking	11	No
11	Constricted visual field		Inferior-temporal quadrant-anopia	NA	NA	Suprasellar	Third ventricle, anterior part of midbrain	Cystic calcified	NA	Left frontal burr hole with endoscopic marsupialization of cyst	48	Yes

In Table 4, we analyzed the associations between age, gender, best corrected visual acuity during presentation, duration of symptoms before treatment, impaired optic nerve function tests, visual field defects, tumor size, type of surgery and recurrence with good visual acuities after the one-year post-operative period. The analysis produced evidence of significant associations between best corrected visual acuity during presentation, duration of symptom prior to consultation, impaired optic nerve function tests, visual field defects and good visual acuity after one-year post-operative period (P < 0.05, Fisher exact test).

**Table 4:** Factors affecting good final visual acuity. *Based on 22 eyes; p-value < 0.05 is statistically significant, Fisher Exact Test. VA: Visual acuity

Variables	Frequency, n (%)	Final visual acuity (6/12 or Better), n (%)	p-value
Age of diagnosis (years)			0.074
Less than six years old	6 (27.3)	5 (22.7)	
Six years old and older	16 (72.7)	6 (27.3)	
Gender			0.33
Male	14 (63.6)	6 (27.3)	
Female	8 (36.4)	5 (22.7)	
VA presentation*			0.04
6/6-6/12	13 (59.1)	9 (40.9)	
Worse than 6/15	9 (40.9)	2 (9.1)	
Duration of symptoms prior to consultation			0.012
Less than 12 months	14 (63.6)	4 (18.2)	
12 months and longer	8 (36.4)	7 (31.8)	
Optic nerve function*			0.04
Impaired	9 (40.9)	2 (9.1)	
Normal	13 (59.1)	9 (40.9)	
Visual field*			0.012
Impaired	14 (63.6)	4 (18.2)	
Normal	8 (36.4)	7 (31.8)	
Imaging findings			0.11
Tumor size (by greatest dimension)			
4 cm and greater	12 (54.5)	9 (69.2)	
Less than 4 cm	10 (45.5)	4 (30.8)	
Treatment			1
Total resection	16 (72.7)	8 (39.4)	
Partial resection/biopsy	2 (9.1)	1 (4.5)	
Refused surgery	4 (18.2)	2 (9.1)	
Recurrence			0.135
Yes	6 (54.5)	4 (18.2)	
No	3 (27.3)	5 (22.7)	
Missing	2 (18.2)	2 (9.1)	

## Discussion

Improved visual outcome is the main concern for ophthalmologists and neurosurgeons managing pediatric craniopharyngioma. Table 5 summarizes published reports on visual presentation and outcome in patients with pediatric craniopharyngioma from Asian countries, the United Kingdom, Spain, Israel, and France, as well as those included in our study [6, 8-13].

**Table 5:** Comparison of published studies on pediatric craniopharyngioma. *Calculated based on 22 eyes in our study. VA: Visual acuity; U: Unavailable; NS: Not specified.

Author (Year)	Our study (2019)	Drimtzias et al. (2014) [6]	Puget et al. (2007) [13]	Mediero et al. (2015) [8]	Lee et al. (2012) [11]	Jung et al. (2010) [12]	Bialer et al. (2012) [9]
Country	Malaysia	United Kingdom	France	Spain	Korea	Korea	Israel
Population							
Adult	0	0	0	0	0	0	0
Children	11	20	66	10	27	17	20
Mean age (SD)	9.5 (4.2)	7.3	7.4	5	U	12	6.5 (3.9)
Gender, n (%)							
Male	7 (63.6)	10 (50.0)	42 (63.6)	6 (60.0)	15 (55.6)	12 (70.6)	10 (50.0)
Female	4 (36.4)	10 (50.0)	24 (36.4)	4 (40.0)	12 (44.4)	5 (16.7)	10 (50.0)
Systemic symptoms, n (%)							
Headache	8 (72.7)	14 (70.0)	U	U	18 (66.7)	13 (76.5)	6 (55.0)
Nausea/vomiting	7 (63.6)	U	U	U	6 (22.2)	NS	U
Motor deficit	2 (18.2)	U	8 (12.0)	U	U	0 (0)	U
Seizure	1 (9.1)	U	3 (4.5)	U	U	0 (0)	1 (9.0)
Ocular symptoms, n (%)							
Visual loss	4 (36.4)	6 (30.0)	29 (44.0)	U	12 (44.4)	4 (23.5)	1 (9.0)
Color vision defect	1 (9.1)	U	U	U	U	0 (0)	U
Diplopia/strabismus	2 (18.2)	U	U	U	4 (14.8)	0 (0)	3 (27.0)
VA presentation*, n (%)							
6/6–6/12	13 (59.0)	19 (47.5)	U	U	33 (61.1)	U	U
6/15–6/60	3 (13.6)	8 (20)	U	U	14 (25.9)	U	U
Worse than 6/60	6 (27.3)	13 (32.5)	U	U	7 (13.0)	U	U
VA final*, n (%)							
6/6–6/12	11 (50.0)	23 (57.5)	U	12 (60)	37 (68.5)	U	U
6/15–6/60	1 (4.5)	4 (10.0)	U	3 (15.0)	13 (24.1)	U	U
Worse than 6/60	10 (45.5)	13 (32.5)	U	5 (25.0)	4 (7.4)	U	U
Fundus examination*, n (%)							
Optic atrophy	11 (50.0)	12 (60.0)	U	15 (75.0)	14 (30.4)	U	17 (42.5)
Papilledema	2 (9.1)	6 (30.0)	U	0 (0)	10 (21.7)	U	6 (15.0)
Normal finding	8 (36.4)	U	U	5 (25.0)	22 (47.8)	U	U
Visual field defect*, n (%)							
Bilateral hemianopia	2 (9.1)	5 (35.7)	10 (15.0)	2 (20.0)	3 (11.1)	U	4 (26.7)
Unilateral hemianopia	3 (13.6)	U	9 (14.0)	2 (20.0)	3 (11.1)	U	3 (20.0)
Central scotoma	3 (13.6)	U	U	0 (0)	2 (7.4)	U	0 (0)
Quadrantanopia	2 (9.1)	U	6 (9.0)	0 (0)	0 (0)	U	1 (6.7)
Others	1 (4.6)	U	11 (17.0)	2 (20.0)	6 (22.2)	U	1 (6.7)
Not performed/available	5 (22.7)	U	13 (20.0)	3 (30.0)	0 (0)	U	0 (0)
Normal visual field	6 (27.3)	4 (28.5)	23 (35.0)	1 (10.0)	13 (48.1)	U	6 (40.0)
Imaging findings, n (%)							
Suprasellar	11 (100.0)	20 (100.0)	U	U	22 (100.0)	10 (58.8)	U
Suprasellar + Intrasellar	0 (0.0)	0 (0)	U	U	0 (0)	7 (41.2)	U
Cystic	7 (63.6)	10 (50.0)	6 (10.0)	U	0 (0)	11 (64.7)	U
Cystic and solid	4 (36.4)	10 (50.0)	U	U	22 (100.0)	6 (35.3)	U
Solid	0 (0.0)	0 (0)	U	U	0 (0)	0 (0)	U
Calcification	5 (45.5)	12 (60.0)	U	U	18 (81.8)	15 (88.2)	U
Hydrocephalus	U	U	35 (53.0)	U	3 (13.6)	U	U
Treatment, n (%)							
Total resection	8 (72.7)	7 (35.0)	33 (50.0)	10 (100.0)	27 (100.0)	12 (70.6)	NS
Partial resection/biopsy	1 (9.1)	12 (60.0)	33 (50.0)	0 (0)	0 (0)	5 (29.4)	NS
No surgery	2 (18.2)	1 (5.0)	0 (0)	0 (0)	0 (0)	0 (0)	NS
Histopathology, n (%)							
Squamous	0 (0)	U	U	U	U	0 (0)	U
Papillary	0 (0)	U	U	U	U	1 (5.9)	U
Adamantinoma	7 (63.6)	U	65 (98.5)	U	U	16 (94.1)	U
Recurrence	6 (54.5)	11 (55.0)	35 (53.0)	2 (20.0)	11 (40.7)	2 (11.8)	U

The mean age of presentation was 9.5 (4.2) years in our study. In the majority of the studies reviewed, the mean age of presentation was less than 10 years [6,8,9,11-13]. In contrast, Jung et al. reported the mean age was 12 years [12]. There was a male preponderance in our study population, which is also consistent with other pediatric craniopharyngioma studies [8,11-13]. In contrast, Drimtzias et al. and Bialer et al. from the United Kingdom and Israel respectively, reported an equal ratio of males and females [6,9].

The most common symptom reported by our patients was headache (72.7%), which is consistent with findings documented by other studies (ranging from 55 to 76.5%) [6,9,10-11]. However, visual loss or blurring of vision was only noted in one-third of our patients (36.4%, four eyes). This observation is also similar to studies from the United Kingdom (30.0%) and Korea (23.5%) [6,12]. A slightly higher percentage was reported from a second study from Korea (44.4%) and France (44.0%) [11,13]. This observation suggests that these children were not able to express or may not have been aware of visual symptoms during the early stages of the disease.

Among our patients, papilledema during presentation was only observed in two eyes (9.1%), while optic atrophy was documented in 11 eyes (50%). This finding is also consistent with studies reported by Drimtzias et al. (30% papilledema, 60% optic atrophy), Mediero et al. (none had papilledema, 75% showed optic atrophy) and Bialer et al. (15% papilledema, 42.5% optic atrophy) [6,8-9]. These data indicate that the diagnosis was only confirmed at a later stage, after the disease had progressed.

We found a significant association between presenting best corrected visual acuity and good final visual outcome (p = 0.040). Nine patients (40.9%) presented with visual acuity worse than or equal to 6/15 (20/50) and, of this group, only two patients (9.1%) documented good final visual acuity (6/12 or 20/40, one year after treatment). A similar pattern was reported by Repka et al. [10]. They evaluated preoperative and postoperative visual acuities in 12 children younger than 18 years with craniopharyngioma. They reported that 17% of their patients had visual acuity worse than 20/60 (6/12) in the better eye at the time of diagnosis, and 27% of their patients continued to have visual acuity worse than 20/40 (6/12) in the better eye postoperatively [13]. In contrast, Drimtzias et al. and Lee and Hwang reported a higher percentage of visual acuity at 20/40 (6/12) after one year (57.5%, 68.5% respectively) [6,11].

We observed a significant association between the duration of systemic symptoms before consultation and good final visual acuity at the one-year post-operative period (p = 0.12). Fourteen patients (63.6%) presented less than one year after the onset of systemic symptoms. Four patients (18.2%) had good final visual acuity after one year. We had difficulty finding published data on this issue for comparison.

However, Chen et al. reported a median duration of systemic symptoms of seven months in their patients [14]. Lee and Hwang documented duration of 5.2 ± 6.8 months in children with craniopharyngioma with normal visual field, and 8.9 ± 10.6 months in those with abnormal visual fields [11]. However, no further analysis regarding the final visual outcome was performed by these authors [11,14].

All patients were examined for optic nerve functions during their first consultation, and at each follow-up visit to detect signs of optic neuropathy. The examination included visual acuity tests, afferent pupillary defect assessment, color vision tests, light brightness and visual field assessments. Impairment of the optic nerve functions identified during diagnosis of craniopharyngioma is significantly associated with final visual acuity after treatment (p = 0.040). Nine patients (41%) showed evidence of optic neuropathy, while 13 patients (59%) showed normal optic nerve function during diagnosis. Only two patients (9.1%) who had impaired optic nerve functions displayed good visual acuity a year after treatment.

We also noted a significant association between visual field defects and final visual acuity (p = 0.012). Fourteen eyes (63.6%) had visual field defects in our series. Only four patients (18.2%) had good visual acuity one year after treatment. Our findings agree with Lee and Hwang who reported that patients with normal visual fields at presentation were more likely to have better visual acuity postoperatively [11].

We found no significant association between the size of the tumor and good visual acuity post operation (p = 0.110). Among our patients, 54.5% had tumor that were larger than 4 cm, as measured on CT scans or MRI findings, based on the greatest dimension. Our findings were similar to Wan et al. who also reported 54% of their patients had tumor sizes greater than 4 cm. Interestingly, Wan et al. also found no significant association between tumor size and visual decline [15]. Lee and Hwang supported these findings, noting that there was no significant difference in tumor size with visual field defects [11].

We observed that six patients (54.5%) had tumor recurrences, which is consistent with other studies that reported a recurrence rate of 40.7% to 55% [6,11,13]. However, there was no significant association observed between recurrence of tumors and final visual acuity.

## Conclusions

Visual presentations in our study were fairly similar to previous reported studies. One-third of our patients presented late with permanent visual loss, and almost half of our patients had significant visual impairment after one-year post-operative period. There were significant associations between presenting visual acuity, duration of symptoms, impairment of optic nerve function tests, and visual field defects during presentation, and final visual acuity at one year after treatment.
